# A Multi-Source Data Fusion Network for Wood Surface Broken Defect Segmentation

**DOI:** 10.3390/s24051635

**Published:** 2024-03-02

**Authors:** Yuhang Zhu, Zhezhuang Xu, Ye Lin, Dan Chen, Zhijie Ai, Hongchuan Zhang

**Affiliations:** College of Electrical Engineering and Automation, Fuzhou University, Fuzhou 350108, China

**Keywords:** multi-source data fusion, wood defect detection, deep learning, U-Net, semantic segmentation

## Abstract

Wood surface broken defects seriously damage the structure of wooden products, these defects have to be detected and eliminated. However, current defect detection methods based on machine vision have difficulty distinguishing the interference, similar to the broken defects, such as stains and mineral lines, and can result in frequent false detections. To address this issue, a multi-source data fusion network based on U-Net is proposed for wood broken defect detection, combining image and depth data, to suppress the interference and achieve complete segmentation of the defects. To efficiently extract various semantic information of defects, an improved ResNet34 is designed to, respectively, generate multi-level features of the image and depth data, in which the depthwise separable convolution (DSC) and dilated convolution (DC) are introduced to decrease the computational expense and feature redundancy. To take full advantages of two types of data, an adaptive interacting fusion module (AIF) is designed to adaptively integrate them, thereby generating accurate feature representation of the broken defects. The experiments demonstrate that the multi-source data fusion network can effectively improve the detection accuracy of wood broken defects and reduce the false detections of interference, such as stains and mineral lines.

## 1. Introduction

In the wood industry, broken defects, such as cracks and dead knots on the wood surface, can destroy the structural integrity of wood and affect the quality of wooden products; therefore, these defects need to be strictly detected and eliminated. These broken defects can be inspected with human vision and touched by experienced workers; however, due to human subjectivity, problems such as low efficiency and poor accuracy remain within the manual inspection. Therefore, it is necessary to develop an automatic broken defect detection method for the wood industry.

Initially, many non-destructive approaches were developed to identify a broken defect region, such as the ultrasonic [1], infrared [2], stress wave [3], and acoustic laser techniques [4], which are sensitive to the depth variation in the broken region, but ignore the appearance characteristic, leading to an incomplete detection of the broken defects. With the support of machine vision, which focuses on image data with high resolution and intuitiveness, the researchers proposed numerous deep-learning-based approaches, which utilize convolutional neural networks (CNN) with excellent feature representation capabilities [5] to perform wood defect detection in region level [6,7] or pixel level [8].

**Figure 1:**
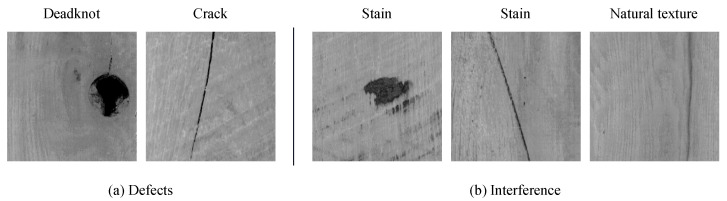
The challenges of wood broken defect detection.

The deep-learning-based approaches have effectively improved the detection accuracy of wood defects. However, these defection methods based on single image data have difficulty when distinguishing the wood broken defects with the interference similar to the defects due to the limitation of feature representation. Specifically, in the wood image data shown in Figure 1, the natural texture and the stains represent similar shapes and colors with the dead knot and crack defects, the subtle differences cause confusion for the model when distinguishing the wood broken defects from the interference, resulting in frequent false detections. In the practical wood industrial field, interference without damage is allowed to be retained, which means that a lot of woods without damage will be wasted during the wood production process.

To address this issue, a multi-source data detection method is proposed for the wood broken defect detection. By introducing the depth information to obtain the discriminative features of wood broken defects, and combining the color texture information to achieve complete segmentation of the broken defects. In addition, a multi-source data fusion network is proposed for the wood broken defect detection based on U-Net [9]. Specifically, the contributions of this article are as follows:

1. Laser profile sensors and cameras are adopted to capture the depth data and image of wooden board. Furthermore, a multi-source data fusion network is designed to recognize the wood broken defect by simultaneously focusing on the depth information and color texture information, to achieve a precise segmentation of the wood broken defects and effectively eliminate the influence of interference;
2. An improved ResNet34 is developed to efficiently extract multi-level features from wood image and depth data, in which the depthwise separable convolution (DSC) and dilated convolution (DC) are added to reduce feature redundancy and enhance the overall perception of the wood broken defects;
3. An adaptive interacting fusion (AIF) module is designed to integrate the features extracted from image and depth data via calculating the weights of different features, achieving accurate feature representation of the broken defect. Additionally, the coordinate attention (CA) is added to further highlight the discriminative effect of depth information.

The rest of this article is structured as follows: Section 2 reviews the related works. The dataset collection process is introduced in Section 3. Section 4 presents the proposed multi-source data fusion model in detail. Section 5 gives the experimental results and the related analysis. The discussions of the potential application and improvements of the proposed method are given in Section 6. The conclusion of this article is given in Section 7.

## 2. Related Works

For wood surface defect detection, the methods mainly include traditional detection approaches and deep-learning-based detection approaches.

### 2.1. Traditional Wood Defect Detection Approaches

Traditional detection approaches can be divided into the following three categories: threshold-based approaches, statistical-based approaches, and model-based approaches. The threshold-based approaches generally combine color difference and mathematical morphology [10], setting a suitable threshold [11] to segment the defective region in the local [12] or global [3] ranges. For the statistical-based approaches, which usually adopt the local binary pattern (LBP) [13,14] and gray level co-occurrence matrix (GLCM) [15] to measure the distribution of pixel values so as to achieve a classification result in the image level. The model-based approaches generally design specific feature vectors according to the color, shape, and texture of defects, and utilize various machine learning algorithms, such as the support vector machine (SVM) [16], the regression tree (CART) [17], and a neural network [18] as the classifiers so as to achieve a satisfactory detection performance on small-scale detection samples.

These approaches have achieved good performance on specific detection scenarios; however, the dependence on expertise limits generalization for application.

### 2.2. Deep-Learning-Based Wood Defect Detection Approaches

In recent years, the convolutional neural network (CNN) is introduced to locate and identify wood surface defects [19,20,21]. To improve the detection accuracy, Tu et al. [6] designed an improved Gaussian YOLOv3 by adding a complete intersection over union (CIoU) loss function to reduce repeated detection. Considering the complicated characteristics and various sizes of wood defects, Meng et al. [22] proposed an improved YOLOv5 model based on a semi-global network (SGN) to generate adequate contextual information of wood defects; furthermore, Zhu et al. [7] proposed an efficient multi-level-feature integration network (EMINet) to extract the discriminative features of defects. Focusing on the tiny cracks, Lin et al. [8] proposed a data-driven semantic segmentation network to recognize cracks at the pixel-level. Due to the limitation of the receptive field of CNN, Ge et al. [23] introduced a detection transformer (DETR) to improve the detection performance. Unlike these methods, based on wood surface depth data, Xu et al. [24] designed an improved Bi-LSTM network to identify the detective lines efficiently.

However, the above methods are conducted based on single image data or other data types, which make it difficult to achieve simultaneous complete detection and suppress the interference.

## 3. System Overview

### 3.1. Data Collection

**Figure 2:**
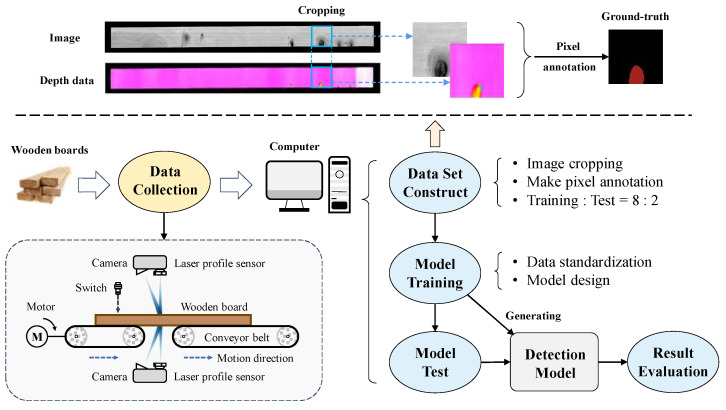
The overall framework of wood broken defect detection process, including data collection platform, dataset constructing, model training, and result evaluation.

The platform used for data collection and defect detection is shown in Figure 2, which is deployed in the industry processing site. The platform is composed of a camera, laser profile sensor, motor, conveyor belt, photoelectric switch, and a computer. The collection process is carried out in an enclosed space, and the linear light sources are deployed inside the space to obtain a stable lighting condition.

Firstly, the wooden board is transported to the data collection area via the conveyor belt. When the wooden board arrives the site of the photoelectric switch, the cameras and laser profile sensors installed at the bottom and top of the conveyor start scanning the wooden board to capture the wood surface image. The obtained data are transmitted to the computer for data processing and defect detection. As shown in Figure 2, the original data are cropped into pieces due to the large aspect of wooden board, and the data with defects are selected to make pixel annotations for model training and testing. It is worth noting that the ground-truth corresponds to the defective region of image data.

### 3.2. Wood Broken Defect Dataset

**Figure 3:**
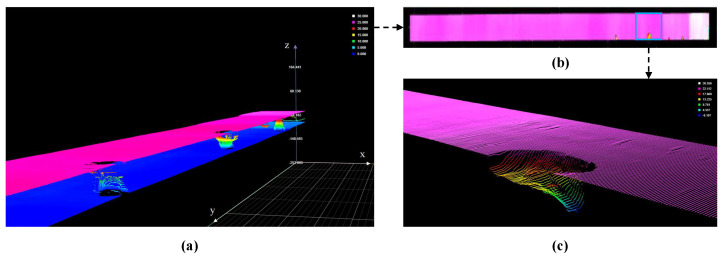
The three-dimensional visualization of wood depth data. (**a**) The 3D visualization of the top and bottom depth data. (**b**) The visualization of the the top depth data. (**c**) The detailed 3D visualization of a wood broken defect.

The obtained data includes image and depth data. The three-dimensional visualization of depth data is shown in Figure 3a, in which the sides with pink and blue denote the top and bottom data, respectively. It can be seen that the distribution of the values are different. Therefore, the z-score is adopted to standardize the value distribution of the top and bottom depth data. In addition, the value of bottom depth data is inverted to be consistent with the top one. Taking a broken defect shown in Figure 3b, its corresponding three-dimensional visualization is shown in Figure 3c. Obviously, there is depression in the broken region of defect.

**Figure 4:**
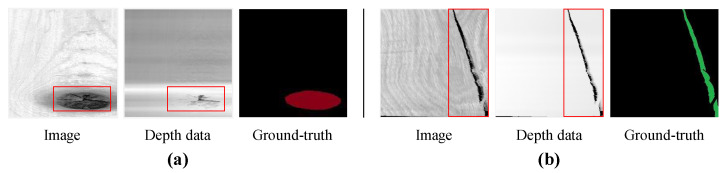
The broken defect data of wood dataset, and the red box denotes the defective region. (**a**) Dead knot. (**b**) Crack.

The broken defects that are detected in this article include dead knots and cracks. The dead knot that generates with the growth of tree refers to a natural defect, it is usually loose or falls off, which forms a cavity and creates a broken area. As shown in Figure 4a, the broken area of a dead knot revealed in the depth data covers a part of the defective region revealed in the image. For the crack shown in Figure 4b, which is formed by the separation of fibers during the growth of wood, the broken area may expand due to exogenic action during transportation. Unlike the dead knot defect, the broken area of crack almost coincides with the defective region.

## 4. Methodology

To obtain the detection results of wood broken defects based on image and depth data, a multi-source data fusion network is proposed based on U-Net, a typical network used for semantic segmentation in various detection scenarios [25,26,27].

### 4.1. Architecture Overview

**Figure 5:**
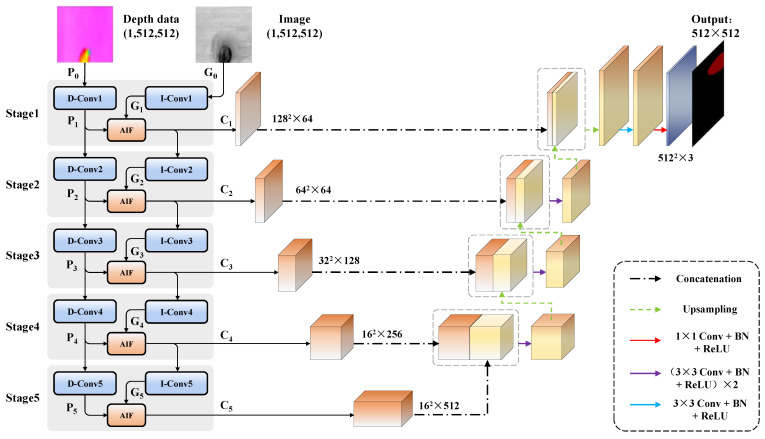
The overall architecture of the proposed multi-source data fusion network, in which the upsampling denotes bilinear upsampling operation, BN denotes batch normalization, and ReLU denotes rectified linear units activation.

The overall architecture of the proposed network is shown in Figure 5, the multi-source data fusion network includes the feature extraction backbone and decoders. Five stages are deployed in the backbone for generating multi-level features $C_{i}$(i∈1,2,3,4,5), and each stage is composed of D-Conv, I-Conv, and the adaptive interacting fusion (AIF) module, in which the D-Conv and I-Conv belong to different branches used for multi-level feature extraction from image and depth data. Considering the critical role of depth variation for recognizing wood broken defects, the branch of image is designed as the auxiliary part of backbone so as to enrich the feature representation of wood broken defects. Specifically, the integrated feature $C_{i}$(i∈1,2,3,4) is used as the input of ${I-\mathrm{Conv}}_{i}$ instead of $G_{i}$ in the next stage, thereby selecting effective color characteristics in various resolutions and enhancing major effect of depth information. The calculation process of feature $C_{i}$ can de performed as follows:

(1) $$\begin{array}{cc} P_{i}={D-\mathrm{Conv}}_{i}\left(P_{i-1}\right),\;\;G_{i} & =\left\{\begin{array}{c} {I-\mathrm{Conv}}_{i}\left(G_{0}\right),\;\;\;\mathrm{if}\;=1\\ {I-\mathrm{Conv}}_{i}\left(C_{i-1}\right),\;\;\mathrm{if}\;=\left\{2,3,4,5\right\} \end{array}\right. \end{array}$$

(2) $$\begin{array}{cc} C_{i} & =\mathrm{AIF}\;(P_{i},G_{i}) \end{array}$$

where $P_{i}$ and $G_{i}$ denote the outputs of ${D-\mathrm{Conv}}_{i}$ and ${I-\mathrm{Conv}}_{i}$, respectively. Finally, the pixel prediction map with size of 512×512×3 is obtained through the decoders stage-by-stage, and the details of decoders is shown in Figure 5.

### 4.2. Backbone

As an essential part of network for extracting various semantic information of object to be detected, the backbone indirectly determines the detection accuracy and speed of network. Numerous current studies focus on improving the accuracy of detection by increasing the scale of network [28,29], resulting in slower detection speed and heavier computing resources, which is difficult to be applied in the practical industry production process. Inspired by the ResNet [30], which is widely used in various industry detection scenarios [31,32] due to its efficient feature extraction capacity, an improved backbone based on ResNet34 is designed to constitute the encoders of the proposed method for efficient feature extraction. The details of the encoders are shown in Table 1, in which the “Number” denotes that the times to repeat the corresponding operation.

**Figure 6:**
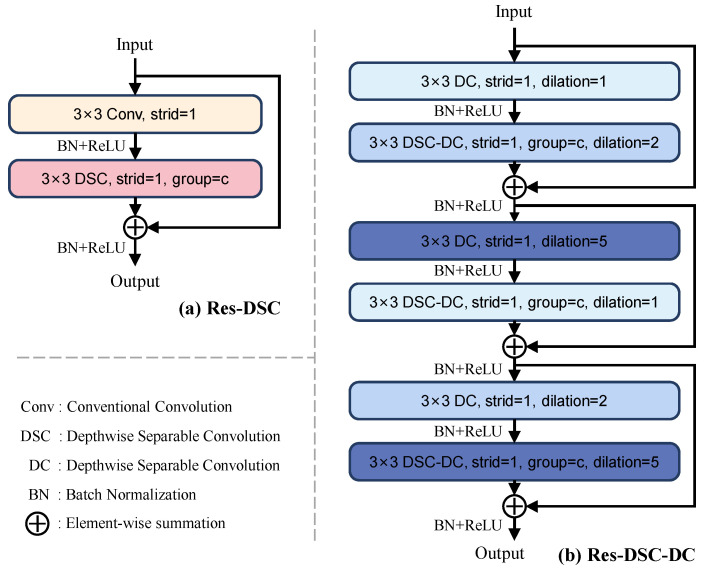
The structures of the proposed Res-DSC and Res-DSC-DC, in which the “group” refers to a hyper-parameter of DSC, the “dilation” refers to the dilation rate of DC.

To reduce feature redundancy and enhance feature extraction capacity of backbone, Res-DSC and Res-DSC-DC are designed to replace the Basicblock of ResNet34, the detailed structure of the two convolution modules are shown in Figure 6. In the backbone, the structure of ${I-\mathrm{Conv}}_{i}$ is the same as ${D-\mathrm{Conv}}_{i}$. Specifically, as shown in Table 1, only the 7 × 7 convolution layer with stride of 2 and 3 × 3 max-pool operation with stride of 2 in the ResNet34 are retained to constitute the stage1 in the encoders; the second and third layers of ResNet34 are replaced with Res-DSC; the fourth and fifth layers are replaced with Res-DSC-DC. Additionally, the 1 × 1 convolution used for downsampling in the shortcut of ResNet34 is removed, and a 3 × 3 max-pool operation with stride of 2 is added to the end of each layer to downsample the feature map so as to further decrease the inference time of model.

#### 4.2.1. Res-DSC

In numerous industrial defect detection scenarios, the depthwise separable convolution (DSC) [33] is widely introduced into the detection networks for reducing the computational resource and feature redundancy [34,35,36], so as to meet the real-time requirement of industry production process. In this article, the DSC is adopted to constitute the designed Res-DSC module.

**Figure 7:**
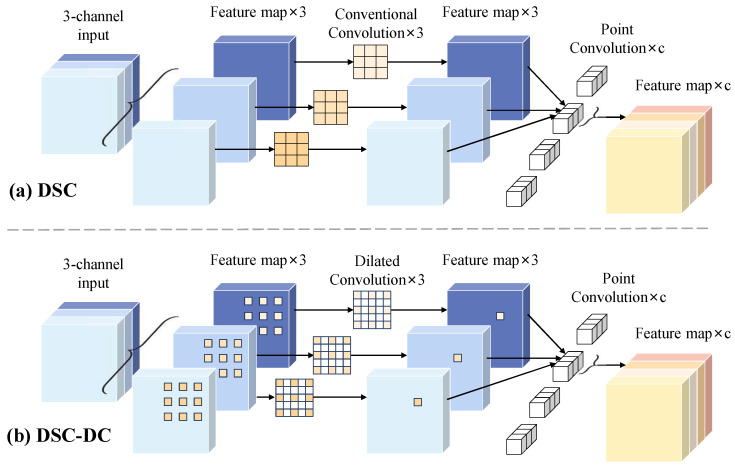
The structures of DSC and DSC-DC, the parameter “c” represents the number of the output channels.

As shown in Figure 7, the DSC is composed of the depthwise convolution and the pointwise convolution. Specifically, each channel of the feature map is firstly calculated by a separate kernel, so as to extract corresponding spatial feature of each channel. Then, the feature maps are integrated by 1 × 1 convolutions, achieving the correlation information among different channels. Compared with the conventional convolution, the DSC can achieve less computational resource, however, it also reduces the diversity of semantic information. Therefore, to optimize the detection network while maintaining the segmentation precision, the conventional convolution and DSC are combined to constitute the Res-DSC module, as shown in Figure 6a, where the detail of parameter “c” is shown in Table 1.

#### 4.2.2. Res-DSC-DC

**Figure 8:**
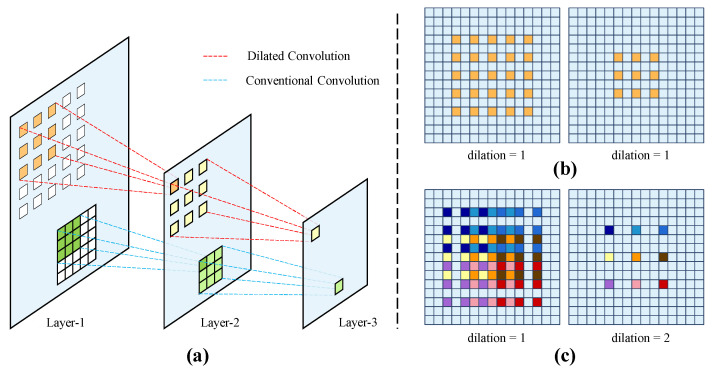
Schematic of receptive field of the dilated convolution and conventional convolution. (**a**) The receptive fields of dilated convolution and conventional convolution. (**b**,**c**) Pixel coverage under different expansion rate groups.

In the CNNs, the receptive field of feature map is expanded with the increase in the number of convolution layers. However, in the ResNet34, the receptive field of conventional convolution is limited [30] for capturing overall perception of wood broken defects with large sizes, thus affecting the classification of defect target. To address this issue, the dilated convolution (DC) [37] is introduced to the backbone to enhance the global context information of network. As shown in Figure 8a, compared with conventional convolution, the DC introduces the dilation rate to obtain a larger receptive field without increasing the parameters of network. To further reduce the computational resource, combining the DSC and DC, the DSC-DC is designed to constitute the Res-DSC-DC module. As shown in Figure 7b, replacing the conventional convolutions in DSC with dilated convolutions, to achieve less parameters.

As shown in Figure 6b, the Res-DSC-DC module is composed of three identical units, and each unit is composed of a DC and a DSC-DC. For the stacking of DC layers, unsuitable expansion rates will lead to gridding issue [38], resulting in feature loss. As shown in Figure 8b, for the continuous DC layers with dilation rates of 1 and 1, the feature map can only realize the semantic information in a checkerboard fashion, and lose a large portion of information, nearly 70%. In contrast, when the dilation rates are set as 1 and 2 (Figure 8c), the coverage area of the pixels can achieve 67%. Therefore, as shown in Figure 6b, the expansion rate of each DCs in the DSC-DC are set as {1, 2, 5, 1, 2, 5} according to [38]. However, the local location information contained in the low-level features of the backbone will be weakened with the increase in receptive field, only the fourth and fifth layers of ResNet34 are replaced with the Res-DSC-DC.

### 4.3. Adaptive Interacting Fusion

**Figure 9:**
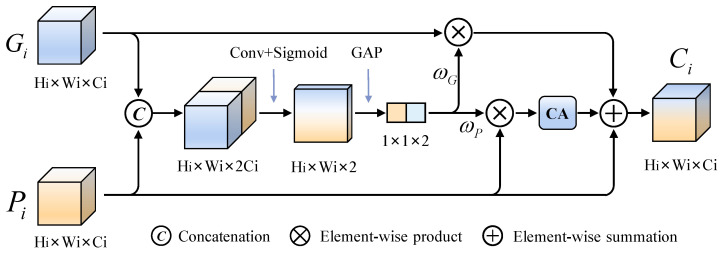
The structure of the proposed adaptive interacting fusion (AIF) module.

For wood broken defect detection, the depth data that can accurately reflect the damaged area are critical to distinguish broken defects. On the other hand, the image data with high resolution contain detailed texture and color information, which can obtain a complete description to the appearance of defects. Thus, the image and depth data are both necessary for achieving precise recognition to the wood broken defects. To take full advantage of them, an adaptive interacting fusion (AIF) module is designed to integrate two feature maps in the encoders, by adaptively calculating the weights of each feature, to achieve accurate representation of broken defects. The detailed structure of AIF module is shown in Figure 9.

Given the $G_{i}\in R^{H_{i}\times W_{i}\times C_{i}}$ and $P_{i}\in R^{H_{i}\times W_{i}\times C_{i}}$ as the inputs of the AIF, the concatenated feature of them is integrated by a 1 × 1 convolution with stride of 1 and followed by a Sigmoid activation function, generating a spatial map with $H_{i}\times W_{i}\times 2$. Going through a global average pool (GAP) function, the weight of each input is obtained and multiplied with the corresponding input. Considering the essential effect of depth data for distinguishing broken defects with interference, a coordinate attention (CA) [39] mechanism is introduced to enhance the location information of the weighted feature $P_{i}$ in spatial dimension and suppress the influence of interference. Finally, the integrated feature $C_{i}\in R^{H_{i}\times W_{i}\times C_{i}}$ is obtained by adding the two weighted features and $P_{i}$ in pixel level. Mathematically, it can be described as follows:

(3) $$\begin{array}{cc} \omega_{G},\omega_{P} & =Split\;(\;GAP\;\left(\;\sigma \;\left(\;W_{1\times 1}*CAT\;\left(G_{i}\;,\;P_{i}\right)\right)\right)) \end{array}$$

(4) $$\begin{array}{cc} C_{i} & =\left(\;\omega_{G}⊗G_{i}\;\right)⊕CA\;\left(\;\omega_{P}⊗P_{i}\;\right)⊕P_{i} \end{array}$$

where Split(·) denotes that the vector with 1×1×2 is split into the weights $\omega_{G}$ and $\omega_{P}$, CAT refers to the concatenation, $W_{1\times 1}$ denotes 1×1 convolution operation, σ refers to the Sigmoid activation function, ⊕ and ⊗, respectively, denote the element-wise summation and production.

### 4.4. Loss Function

To improve the detection accuracy, the hybrid loss is introduced to supervise the training process. Formally, the total loss is defined as follows:

(5) $$\begin{array}{cc} L=\; & L_{ce}\;+\;L_{dice} \end{array}$$

where the $L_{ce}$ and $L_{dice}$, respectively, denote the cross-entropy (CE) loss and dice loss [40], which focus on the local pixel loss and global loss. This combination is effective for achieving the complete segmentation result of broken defects.

Specifically, dice loss is adopted to measure the similarity between ground-truth and predicted map. The value range of dice loss is limited between 0 and 1. It can be defined as:

(6) $$\begin{array}{cc} L_{dice}=\;1 & -\frac{2\sum_{i}^{H\times W}P_{i}G_{i}+\epsilon }{\sum_{i}^{H\times W}{\left(P_{i}\right)}^{2}+\sum_{i}^{H\times W}{\left(G_{i}\right)}^{2}+\epsilon } \end{array}$$

where $P_{i}$ and $G_{i}$ refer to each pixel of the segmentation prediction map and the corresponding ground-truth, the H and W, respectively, denote the height and width of the input data, ϵ denotes the smoothing item to avoid zero division.

## 5. Experiments

### 5.1. Implementation Details

The wood broken defect dataset with image and depth data is provided by a third-party partner company. To demonstrate the effectiveness of the proposed method, a series of experiments are carried out on the wood dataset. The dataset totally contains 1100 sets of data with pixel-level annotations, each set is composed of an image and a depth data. The dataset is divided into a training set and a test set according to the ratio of 8:2, generating a total of 880 sets of training data and 220 sets of test data.

The image and depth data is resized to 512×512 for training and testing. For the training of network, the number of epochs is set as 200 and the batch size is set as 6. During the training process, the data are randomly selected as the inputs, and the Adam optimization technique is selected to optimize the parameters of the network. The learning rate is set as 0.0001 initially, and adjusted according to the cosine annealing learning rate scheduling. The details of the environment used for conducting the experiments are listed in Table 2.

### 5.2. Evaluation Metrics

The pixel accuracy (Acc), mIoU, mean precision (Pre), mean recall (Rec), and mF1 are generally adopted to evaluate the performance of semantic segmentation tasks. The pixel accuracy denotes the ratio of pixels that are correctly classified. mIoU represents the averaged overlapping ratio between the ground-truth and the predicted map. The above metrics are formulated as follows:

(7) $$\begin{array}{cc} \mathrm{Accuracy} & =\frac{\sum_{i=1}^{N}{\mathrm{TP}}_{i}}{\sum_{i=1}^{N}{\mathrm{TP}}_{i}+\sum_{i=1}^{N}{\mathrm{FP}}_{i}} \end{array}$$

(8) $$\begin{array}{cc} \mathrm{mIoU}=\frac{1}{N} & \sum_{i=1}^{N}\frac{p_{ii}}{\sum_{j=1}^{N}p_{ij}+\sum_{j=1}^{N}\left(p_{ji}-p_{ii}\right)} \end{array}$$

(9) $$\begin{array}{cc} \mathrm{mPre} & =\frac{1}{N}\sum_{i=1}^{N}\frac{{\mathrm{TP}}_{i}}{{\mathrm{TP}}_{i}+{\mathrm{FP}}_{i}} \end{array}$$

(10) $$\begin{array}{cc} \mathrm{mRec} & =\frac{1}{N}\sum_{i=1}^{N}\frac{{\mathrm{TP}}_{i}}{{\mathrm{TP}}_{i}+{\mathrm{FN}}_{i}} \end{array}$$

(11) $$\begin{array}{cc} \mathrm{mF}1 & =\frac{1}{N}\sum_{i=1}^{N}\frac{2\times {\mathrm{Pre}}_{i}\times {\mathrm{Rec}}_{i}}{{\mathrm{Pre}}_{i}+{\mathrm{Rec}}_{i}} \end{array}$$

where *N* equals to the number of defect categories plus one, and this category denotes the background, $p_{ij}$ denotes the number of class-*i* pixels that are classified as class-*j*, TP denotes the number of pixels that are correctly classified, FP denotes the number of negative pixels that are misclassified as positive samples, and FN denotes the number of positive pixels that are misclassified as negative samples. Considering about the deployment of detection model in industry, floating point operations per second (FLOPs) and parameters are utilized to measure the computational expense.

### 5.3. Experiments on the Selection of Backbone

**Figure 10:**
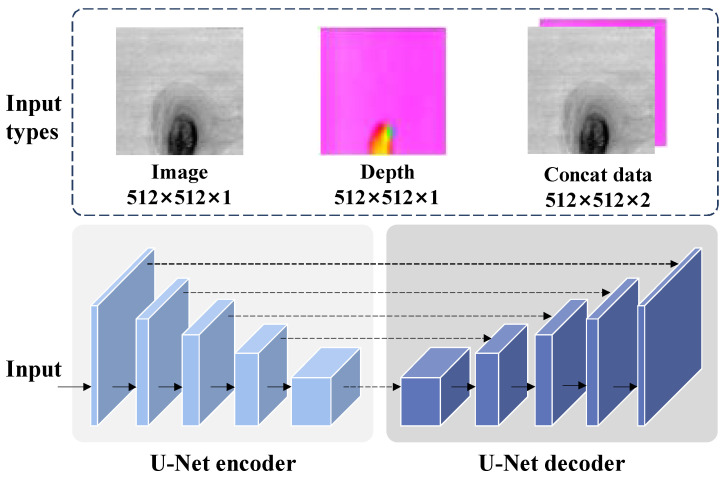
The schematic of different input types.

In a practical industrial production process, the defect detection model needs to simultaneously meet the requirements of detection accuracy and speed. Thus, to select an efficient backbone as the encoder of segmentation network, a series of experiments based on ResNet with different scales (ResNet18, ResNet34 and ResNet50) were carried out on our wood dataset. As shown in Figure 10, the U-Net was adopted as the baseline network by training the network with three types of inputs, including image, depth data, and the concatenated data with a size of 512×512×2, to demonstrate the rationality of the backbone selection; the experimental results are shown in Table 3. Additionally, the parameters and computational resources of the three backbones are shown in Table 4. As can be seen, compared with the detection models using ResNet18 and ResNet50 as the encoder, the model using ResNet34 has achieved the best mIoU and mF1 on the three types of input data. For the ResNet18 backbone with least parameters of 11.17M and FLOPs of 0.93 × $10^{9}$, its detection performance is not enough for application in industry; therefore, ResNet34 backbone was selected as the basic encoder of segmentation network.

It is worth noting that, compared with the ResNet50, which has more parameters, the model that used ResNet34 achieved a better performance. This situation can be explained that the network with larger scale generates redundant information, resulting in poor detection results. In addition, compared with the model using single image input, the concatenated data have achieved worse detection performance, which shows that utilizing the direct concatenation of the depth data and image as the input will cause confusion for the model to achieve accurate description of broken defects.

### 5.4. Ablation Study

To demonstrate the effectiveness of the improvements in the ResNet34 backbone, the ablation experiments of DSC and DC were carried out on the wood dataset, and the experimental results are shown in Table 5. In addition, the parameters and computational resources of the improved ResNet34 are shown in Table 6.

According to the analysis in Section 5.3, ResNet34 was selected as the encoder of the proposed network. As can be seen, when adding the DSC and DC to the backbone, the mIoUs were improved by 0.29% and 0.36%, respectively, which shows that these improvements are effective for the improvement in the segmentation of wood broken defects. It is worth noting that the DSC with less parameters achieved better performance than the ResNet34 without DSC, which demonstrates that the DSC is effective for the reduction in feature redundancy and impairs the influence of interference. When adding DSC and DC together to the backbone, compared with the baseline network, the mIoU was improved by 1.60% and mF1 was improved by 1.12%, while the parameters and FLOPs are decreased between 10.00 M and $0.94\times 10^{9}$, which is almost the same as ResNet18. In summary, the improved ResNet34 can make the model achieve a better detection performance with less computational expenses, which is more suitable for the deployment.

### 5.5. Experiments on Fusion Methods

In this paper, multi-source data are used to detect the wood surface broken defects, and the AIF module is designed to integrate the two features generated from depth data and image. In order to verify the effectiveness of adopting multi-source data to perform wood defect detection, based on the improved ResNet34, the experiments with single data were carried out on the wood dataset, including single depth data and single image. The experimental results are shown in Table 7, in which the third row denotes our proposed method. Compared with the method with single depth data, the proposed method has improved the mIoU and mF1 by 7.38% and 4.71%, respectively, which shows the limitation of depth data for capturing color characteristics. Compared with the model with single image, the proposed method has improved the mIoU and mF1 by 2.00% and 1.25%, respectively. Benefiting from the depth information, the model can effectively suppress the influence of interference.

**Figure 11:**
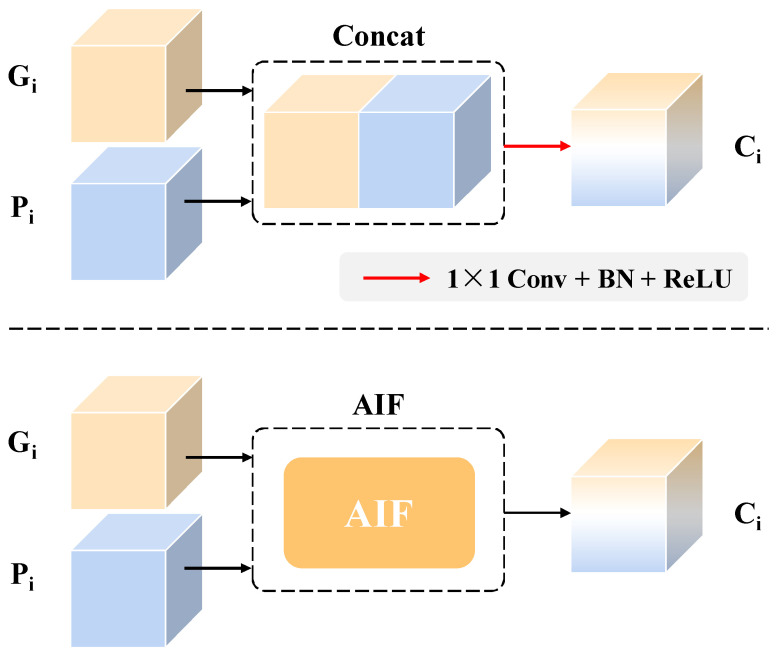
Different fusion types between $G_{i}$ and $P_{i}$. The upper type denotes the concatenation operation, and the lower type refers to our proposed method.

In addition, to verify the effectiveness of AIF module, the experiment of another fusion type between $P_{i}$ and $G_{i}$ was conducted. As shown in Figure 11, the concatenated feature $P_{i}$ and $G_{i}$ is integrated by an 1×1 convolution with batch normalization (BN) and rectified linear units (ReLU) activation. In contrast, the proposed fusion type has improved the mIoU and mF1 by 1.17% and 0.80%, respectively, which demonstrates the effectiveness of AIF module.

### 5.6. Comparisons of Different Methods

To demonstrate the effectiveness of the proposed multi-source data fusion detection method, the experiments of various segmentation methods based on single image data were carried out for comparison, including U-Net [9], PSPNet [41], DeepLabv3 [42], and SegFormer [43]. To achieve a fair comparison, all the methods, including the proposed method, adopted the pre-trained model to initialize the parameters of the network and were retrained on the wood dataset. The experimental results are shown in Table 8. Obviously, the proposed method has achieved the best mIoU and mF1 of 79.73% and 88.11%. Especially for the mean precision, the proposed method has achieved 88.86%, which means that a lower false drop rate for the interference. For the U-Net with VGG16, which has achieved the best mean precision of 89.57%, the mean recall is 2.56% lower than the proposed method.

**Figure 12:**
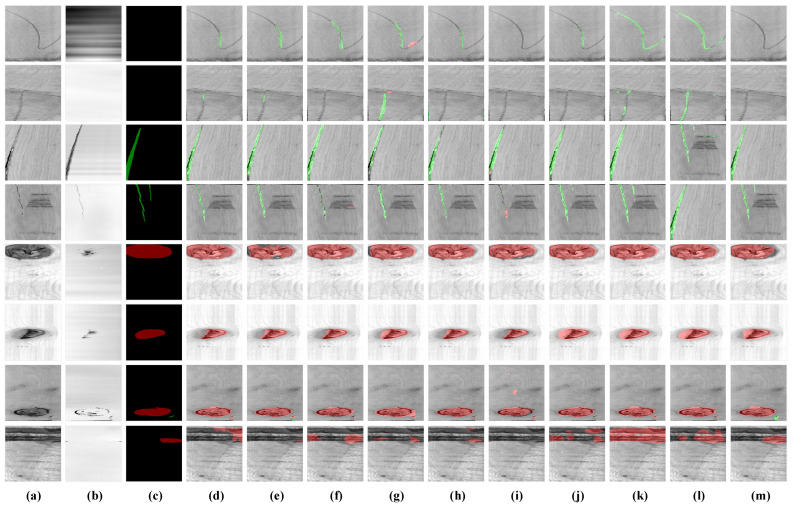
Comparison of segmentation results. (**a**) Original image. (**b**) Original depth data. (**c**) Ground-truth. (**d**) U-Net with ResNet50. (**e**) U-Net with VGG16. (**f**) PSPNet with ResNet50. (**g**) PSPNet with MobileNetv2. (**h**) DeepLabv3 with MobileNetv2. (**i**) DeepLabv3 with Xception. (**j**) SegFormer with MiT-B0. (**k**) SegFormer with MiT-B1. (**l**) SegFormer with MiT-B2. (**m**) Ours.

In addition, to highlight the advantages of the proposed multi-source data fusion detection method for distinguishing the broken defects with the interference, the detection results of the crack, dead knot, and interference are visualized in Figure 12. For the interference shown in the first, second, and last rows, the other methods with single image had difficulty distinguishing the interference with the broken defects, resulting in false detections. In contrast, the proposed method has effectively eliminated the interference. For the crack defects shown in third and forth rows, false detections occurred with the other methods, while the proposed method achieved more accurate and complete segmentation results. For the SegFormer, which has achieved complete segmentation results with the defective regions of dead knot defects shown in the fifth and sixth rows; however, it almost failed to distinguish the interference in the images in the first and second rows.

In summary, the multi-source data fusion detection model based on U-Net proposed in this paper can effectively overcome the influence of wood surface interference, and achieve accurate classification and location of wood defects.

## 6. Discussion

### 6.1. Discussion on The Potential Improvement

**Figure 13:**
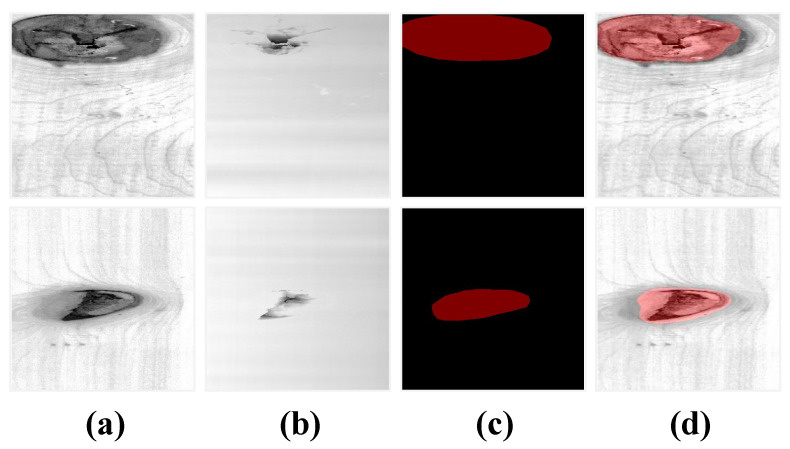
Analysis of failure cases. (**a**) Original image. (**b**) Original depth data. (**c**) Ground-truth. (**d**) Detection result.

The experimental results show that the proposed method outperforms the detection methods with single data. However, some issues still remain in our method. As shown in Figure 13, it can be seen that the proposed method has a lack of integrity in segmenting the defect area of partial defects. Due to the various sizes and shapes of the wood defects, the insufficient dataset makes it difficult to help the detection model learn the complete characteristics of the defects, leading to poor detection results with partial defects. In addition, the difficulty present in making accurate annotations of multi-source data poses a challenge when training the detection model; therefore, the improvements which focus on the dataset is our work in the future.

### 6.2. Discussion on The Practical Application

In this article, the multi-source data fusion network is proposed to accurately recognize wood broken defect and suppress the influence of various interferences on the surface, improving the robustness of the detection model under a complicated industrial environment. The method that combines the depth data and image data is conducive to control the quality of industrial products, such as the classification task for 3D objects and the defect detection task for mechanical workpieces with various shapes. In addition, through the depth data and image data, the engineers in the industrial site can obtain a more intuitive understanding of complex industrial scenes.

## 7. Conclusions

In this paper, a multi-source data fusion network is proposed for the wood surface broken defect detection based on U-Net, by combining image and depth data to suppress the influence of interferences, such as stains and mineral lines, on the wood surface and achieve an accurate segmentation of broken defects. Firstly, an improved ResNet34 is designed to efficiently extract the multi-level features of wood image and depth data. Specifically, the depthwise separable convolution (DSC) and dilated convolution (DC) are added into the backbone to decrease the computational expense and feature redundancy. To achieve an accurate feature representation of wood broken defects, the adaptive interacting fusion (AIF) module is designed to integrate two types of data by calculating the weights of them in the channel dimension; thus, obtaining the integrated features. The experiments show that the proposed method can achieve accurate segmentation results with less parameters and effectively reduce the false detection of the interference.

## Figures and Tables

**Table 1:** The detail structure of the backbone.

Backbone	Type	Number	Output Size
Input	–	–	512 × 512 × 1
D-Conv1 & I-Conv1	7 × 7 Conv, ^1^s = 2	1	256 × 256 × 64
	3 × 3 Max-pool, s = 2	–	128 × 128 × 64
D-Conv2 & I-Conv2	Res-DSC, ^2^c = 64	3	128 × 128 × 64
	3 × 3 Max-pool, s = 2	–	64 × 64 × 64
D-Conv3 & I-Conv3	Res-DSC, c = 128	4	64 × 64 × 128
	3 × 3 Max-pool, s = 2	–	32 × 32 × 128
D-Conv4 & I-Conv4	Res-DSC-DC, c = 256	2	32 × 32 × 256
	3 × 3 Max-pool, s = 2	–	16 × 16 × 256
D-Conv5 & I-Conv5	Res-DSC-DC, c = 512	1	16 × 16 × 512

^1^ The parameter “s” denotes the stride of the convolution. ^2^ The parameter “c” denotes the number of group of the DSC in Figure 6.

**Table 2:** Experimental environment.

Category	Version
GPU	Nvidia GTX 2060S (12 GB, 1470 MHz) (Nvidia, Clara, CA, USA)
CPU	Intel i5-12400F (2.5 GHz 4.4 GHz) (Intel, Clara, CA, USA)
Programming	Python 3.8.8 + Pytorch 1.10.0 + Cuda 102
Operating system	Windows 11 × 64

**Table 3:** Performance comparison of different backbones and different input types.

Input Types	U-Net Encoder	mIoU (%)	Acc (%)	mRec (%)	mPre (%)	mF1 (%)
Depth Data	ResNet18	69.06	97.97	82.06	78.20	80.08
	ResNet34	**71.32**	**98.05**	82.03	**81.37**	**81.70**
	ResNet50	70.65	97.97	**84.06**	78.67	81.28
Image	ResNet18	76.85	98.53	**88.56**	84.01	86.23
	ResNet34	**77.19**	**98.64**	85.81	**86.87**	**86.34**
	ResNet50	76.52	98.53	87.99	83.97	85.94
^1^ Concat data	ResNet18	76.79	98.60	85.59	86.51	86.05
	ResNet34	**76.96**	98.58	**87.56**	84.87	**86.19**
	ResNet50	76.13	**98.65**	83.46	**88.60**	85.95

^1^ The Concat data denotes that the depth data and image are concatenated as the input, as shown in Figure 10. *Note*: Under the current evaluation index, the bold text indicates that the corresponding model has the best effect.

**Table 4:** The parameters and computational resources of different backbone.

Backbone	Parameters (M)	FLOPs
ResNet18	11.17	0.93 × $10^{9}$
ResNet34	21.28	1.90 × $10^{9}$
ResNet50	23.50	2.12 × $10^{9}$

**Table 5:** Ablation study of DSC and DC in the improved ResNet34.

ResNet34	DSC	DC	mIoU	Acc	mRec	mPre	mF1
*√*			76.96	98.58	87.56	84.87	86.19
*√*	*√*		77.25	98.67	85.02	87.82	86.39
*√*		*√*	77.32	98.58	89.40	83.78	86.50
*√*	*√*	*√*	78.56	98.76	86.75	87.88	87.31

**Table 6:** The parameters and computational resources of different backbone.

Backbone	Parameters (M)	FLOPs
ResNet18	11.17	0.93 × $10^{9}$
ResNet34	21.28	1.90 × $10^{9}$
Improved ResNet34 (ours)	10.00	0.94 × $10^{9}$

**Table 7:** Performance comparison of different input data and different fusion types of $P_{i}$ and $G_{i}$.

U-Net Encoder	Data	Fusion Type	mIoU	Acc	mRec	mPre	mF1
Improved ResNet34	Depth	–	72.35	98.19	85.02	81.84	83.40
	Image	–	77.73	98.64	**87.60**	86.13	86.86
	Depth & Image	**AIF** (ours)	**79.73**	**98.85**	87.38	**88.86**	**88.11**
		**^1^ Concat**	78.56	98.76	86.75	87.88	87.31

^1^ The Concat in above table denotes the fusion type of concatenation shown in Figure 11. *Note*: Under the current evaluation index, the bold text indicates that the corresponding model has the best effect.

**Table 8:** Performance comparison of different segmentation methods.

Methods	Backbone	mIoU (%)	Acc (%)	mRec (%)	mPre (%)	mF1 (%)
U-Net	ResNet50	77.70	98.64	87.14	86.89	86.70
U-Net	VGG16	77.84	98.74	84.82	**89.57**	86.79
PSPNet	ResNet50	76.44	98.59	86.56	85.12	85.78
PSPNet	MobileNetv2	73.61	98.36	84.62	82.78	83.68
DeepLabv3	MobileNetv2	75.86	98.59	84.87	85.87	85.36
DeepLabv3	Xception	71.04	98.44	76.99	88.22	76.99
Segformer	MiT-B0	78.91	98.71	89.02	86.38	87.55
Segformer	MiT-B1	77.13	98.48	**90.81**	82.79	86.28
Segformer	MiT-B2	76.98	98.52	88.66	84.27	86.17
Ours	Improve ResNet34	**79.73**	**98.85**	87.38	88.86	**88.11**

*Note*: Under the current evaluation index, the bold text indicates that the corresponding model has the best effect.

## Data Availability

The data cannot be made publicly available upon publication because they are owned by a third party and the terms of use prevent public distribution. The data that support the findings of this study are available upon reasonable request from the authors.

## Abbreviations

The following abbreviations are used in this manuscript:

ResNet	Residual network
DSC	Depthwise separable convolution
DC	Dilated convolution
AIF	Adaptive interacting fusion
CNN	Convolutional neural networks
CA	Coordinate attention
BN	Batch normalization and
ReLU	ReLU denotes rectified linear units
GAP	Global average pool
mIoU	Mean intersection over union
